# Different knockout genotypes of *OsIAA23* in rice using CRISPR/Cas9 generating different phenotypes

**DOI:** 10.1007/s11103-019-00871-5

**Published:** 2019-04-19

**Authors:** Mengmeng Jiang, Huaying Hu, Jing Kai, Milton Brian Traw, Sihai Yang, Xiaohui Zhang

**Affiliations:** State Key Laboratory of Pharmaceutical Biotechnology, School of Life Sciences, Nanjing University, Nanjing, 210023 China

**Keywords:** *Oryza sativa*, *OsIAA23*, CRISPR/Cas9, Root development, Alternative splicing

## Abstract

**Key message:**

We have isolated several *Osiaa23* rice mutants with different knockout genotypes, resulting in different phenotypes, which suggested that different genetic backgrounds or mutation types influence gene function.

**Abstract:**

The Auxin/Indole-3-Acetic Acid (Aux/IAA) gene family performs critical roles in auxin signal transduction in plants. In rice, the gene *OsIAA23* (*Os06t0597000*) is known to affect development of roots and shoots, but previous knockouts in *OsIAA23* have been sterile and difficult for research continuously. Here, we isolate new *Osiaa23* mutants using the CRISPR/Cas9 system in *japonica* (Wuyunjing24) and *indica* (Kasalath) rice, with extensive genome re-sequencing to confirm the absence of off-target effects. In Kasalath, mutants with a 13-amino acid deletion showed profoundly greater dwarfing, lateral root developmental disorder, and fertility deficiency, relative to mutants with a single amino acid deletion, demonstrating that those 13 amino acids in Kasalath are essential to gene function. In Wuyunjing24, we predicted that mutants with a single base-pair frameshift insertion would experience premature termination and strong phenotypic defects, but instead these lines exhibited negligible phenotypic difference and normal fertility. Through RNA-seq, we show here that new mosaic transcripts of *OsIAA23* were produced de novo, which circumvented the premature termination and thereby preserved the wild-type phenotype. This finding is a notable demonstration in plants that mutants can mask loss of function CRISPR/Cas9 editing of the target gene through de novo changes in alternative splicing.

**Electronic supplementary material:**

The online version of this article (10.1007/s11103-019-00871-5) contains supplementary material, which is available to authorized users.

## Introduction

Auxins were the first major phytohormones discovered and orchestrate cardinal plant developmental processes, such as cell elongation, cell division and differentiation, tropic responses to light and gravity, general root and shoot architecture, organ patterning, vascular development, growth in tissue culture, and apical dominance (Woodward and Bartel 2005; Teale et al. 2006). Auxin signal transduction is controlled by interaction between auxin/indole-3-acetic acid (Aux/IAA) and auxin response factor (ARF) proteins (Liscum and Reed 2002). When cellular auxin levels are low, Aux/IAA proteins are stable and form heterodimers with ARF proteins, thereby repressing expression of the target auxin response genes. Elevated cellular auxin levels activate the auxin receptor, transport inhibitor response 1/auxin signaling F-box protein (TIR1/AFB), leading to ubiquitylation and degradation of Aux/IAA proteins through the SCFTIR1/AFBs complex, thereby releasing ARF proteins to activate auxin response genes (Luo et al. 2015).

The *Aux/IAA* gene family members encode short-lived nuclear proteins characterized by four highly conserved domains, designated as domains I, II, III, and IV (Mukesh et al. 2006). Domain I harbors an amino-terminal leucine repeat motif (LxLxLx) and functions as a transcriptional repressor of downstream auxin-regulated genes (Tiwari et al. 2004). Domain II contains a conserved TIR1/AFB recognition sequence, GWPPV, and plays a major role in the stability of Aux/IAA proteins. Mutations in the GWPPV recognition sequence block rapid degradation of Aux/IAAs, thus disturbing the auxin signaling pathway (Worley et al. 2000; Ni et al. 2011). ARF proteins also harbor Domains III and IV, which are involved in homo- and heterodimerization of Aux/IAA and/or ARF proteins (Ouellet et al. 2001).

The plant Aux/IAA family is also large and diverse, consisting of 29 and 31 members in *Arabidopsis thaliana* and rice, respectively (Liscum and Reed 2002; Mukesh et al. 2006). The Aux/IAA gene family has also been identified in other plant species such as *Populus*, maize, potato, grape, soybean, and *Brassica* (Çakir et al. 2012; Ludwig et al. 2013; Singh and Jain 2015; Gao et al. 2016; Li et al. 2017). In *Arabidopsis*, several gain-of-function dominant or semi-dominant mutants of *Aux/IAA* genes have been characterized and found to be related to curl patterns in leaves, light and gravitropic responses, and plant length, particularly lateral root formation (Reed 2001; Overvoorde et al. 2005). Most of these lines have amino acid mutations occur within the core sequence of Domain II. For example, the *Arabidopsis* mutants *iaa3* (Tian et al. 2002), *iaa7* (Mai et al. 2011), *iaa17* (Ouellet et al. 2001), *iaa19* (Tatematsu et al. 2004), *iaa28* (Rogg et al. 2001), and *iaa14* (Fukaki et al. 2002, 2005) show varying degrees of lateral root developmental defects. On the other hand, single T-DNA insertion mutants in 12 of the 29 *Aux/IAA* family members and double or triple mutants of closely related *Aux/IAA* genes show no visible developmental defects compared to the wild-type, suggesting extensive functional redundancy among members of the *Aux/IAA* gene family (Overvoorde et al. 2005). In rice, overexpression and mutant analysis are the major methods of *Aux/IAA* gene function research. Overexpression of *OsIAA4* leads to dwarfism, increased tiller angles, and reduced gravity response (Song and Xu 2013). *OsIAA6* is found to be induced by drought stress and related to tiller outgrowth (Jung et al. 2015). The mutants *Osiaa3*, *Osiaa11*, and *Osiaa13* also show lateral root developmental defects, similar to *Arabidopsis* mutants (Nakamura et al. 2006; Zhu et al. 2012; Kitomi et al. 2012; Zhang et al. 2018). Ni et al. found that the *OsIAA23* gene plays an important role in post-embryonic maintenance of the quiescent center in rice by a semi-dominant mutant (Ni et al. 2011, 2014). This mutant harbored an amino acid mutation within the core sequence of Domain II, which causes “GWPPV” to become “EWPPV”.

Except for the genes mentioned above, the function of most *Aux/IAA* genes in rice has not been elucidated. Because of functional redundancy of the *Aux/IAA* genes, the traditional methods (e.g. T-DNA insertions, knockdowns and point mutations) have yielded few mutants with visible defective phenotypes. Currently, non-synonymous mutations of the core sequence of Domain II are the most effective way to study the *Aux/IAA* genes. However, it is generally difficult to generate mutants with expected mutations via traditional artificial mutagenesis. The clustered regulatory interspaced short palindromic repeat/CRISPR-associated protein 9 (CRISPR/Cas9) gene editing technology is an effective approach that solves this problem (Miao et al. 2013). In addition, mutations within the core sequence GWPPV of Domain II often cause significant developmental defects. Indeed, *Osiaa23* homologous mutants show extremely low levels of fertility, making it inconvenient to preserve mutants and thus hindering subsequent studies on *OsIAA23* (Ni et al. 2011). Kepinski and Leyser (2004) reported that a short sequence of 17 amino acids (AKAQVVGWPPVRNYRKN) around the core sequence of Domain II is sufficient for auxin-regulated interaction. Therefore, we aimed to obtain mutants with reduced developmental defects in rice by site-directed mutagenesis of amino acids around the core sequence GWPPV using the CRISPR/Cas9 system.

In this research, we analyzed *IAA* homologs in the rice genome and selected *OsIAA23* (*Os06t0597000*) as the editing target. We adopted a standard method for the CRISPR/Cas9 system tailored to rice (Miao et al. 2013), and applied it to two rice varieties, Kasalath (*indica*) and Wuyunjing24 (*japonica*). To identify off-target effects and confirm accuracy of the targeted changes, we re-sequenced the mutant lines and screened for mutations in a genome-wide scale, with the result that no other effective mutations were found. Similar to the *Osiaa23* mutant reported by Ni et al. (2011), the Kasalath mutants with a 13 amino-acid deletion downstream of the core sequence of Domain II also showed dwarfing, lateral root developmental disorders, and fertility deficiencies. However, the Wuyunjing24 mutants with a single base-pair insertion downstream of the core sequence of Domain II showed a weak mutant phenotype, yet exhibited normal fertility. Analysis of RNA sequencing data revealed new transcript isoforms in the Wuyunjing24 mutant, which are predicted to produce functional OsIAA23 proteins. The alternative splicing mechanism thus apparently works in the Wuyunjing24 mutant, resulting in the synthesis of a functional IAA protein. Further expression analysis suggests that the recovered *OsIAA23* interacts with its two homologs, *OsIAA1* and *OsIAA15,* especially *OsIAA15,* in a same pathway, thereby preserving the wild-type phenotypes.

## Materials and methods

### Sequence analysis of the OsIAA23 homologs in the rice genome

The *IAA* genes used in this study were as identified by Mukesh et al. (2006). The CDSs of the *IAA* genes were first translated into amino acid sequences and aligned using MUSCLE as implemented in MEGA7 (Kumar et al. 2016). The alignments were used to construct a neighbor-joining tree with a Kimura 2-parameter model. The nonsynonymous (*Ka*) and synonymous (*Ks*) nucleotide substitutions were calculated based on the Nei–Gojobori method. Nucleotide diversity (π) was estimated with Jukes and Cantor correction. Tajima’s D was calculated with a default parameter. The genetic parameters were calculated using a Perl script. The rice genome sequences were supplied by Yuan et al. (2017).

### Rice materials and growth conditions

Two rice cultivars, namely, Kasalath (*Oryza sativa* L. ssp. *indica* cv. Kasalath) and Wuyunjing24 (*O. sativa* L. ssp. *japonica* cv. Wuyunjing24) were selected as the recipient materials. All rice plants, including wild-type and various mutants, were grown in parallel in nutrient-rich soil outdoors within a temperature range of 20–30 °C.

### PAM sequence design and vector construction

The vectors pOs-sgRNA and pH-Ubi-cas9-7 were obtained from Professor Lijia Qu (State Key Laboratory for Protein and Plant Gene Research, Peking-Tsinghua Center for Life Science, College of Life Sciences, Peking University, Beijing, China). The sgRNA guide sequence was designed for targeting *OsIAA23* using CRISPR-P (http://crispr.hzau.edu.cn/CRISPR2/). Default parameters were used to design the guide sequences. Then, the target sequences were checked in the scaffold sequences of Kasalath and Wuyunjing24. An sgRNA situated within Domain II of the *OsIAA23* gene was selected. To clone the spacer, two oligos were synthesized and the 5′ ends of the forward and reverse strands were respectively linked to ‘ggca’ and ‘aaac’ sequences. The primer sequences containing spacers are listed in Supplementary Table S1. After annealing the oligos, the spacer with overhanging sequences was inserted into the *Bsa*I site of the pOs-sgRNA vector. A Gateway Cloning LR reaction (Invitrogen) of the resulting pOs-sgRNA construct was performed with the pH-Ubi-cas9-7 vector to generate the *OsIAA23*-targeting sgRNA:Cas9. After the spacer sequence was verified by Sanger sequencing, the construct was transfected into *Agrobacterium tumefaciens* EHA105 cells.

### Stable transformation and mutant screening

Following a standard approach (Zhang et al. 2015), we transformed the sgRNA:Cas9 vector into two rice varieties (Kasalath and Wuyunjing24) via *Agrobacterium*-mediated transformation. For each recipient rice variety, at least eight flasks, each containing 10 shell-less seeds were set up for callus tissue culture. During *Agrobacterium* infection, callus tissues from one or two flasks were transferred to a single plate. A total of 10 plates of callus tissues were prepared for inoculation with *Agrobacterium*, and at least two transformants from each plate were selected for transplantation. Therefore, at least 20 transformants were developed from different callus lines. Here, independent transgenic events were isolated in the presence of hygromycin B.

For each transformed plant, DNA was extracted from the leaves using the cetyl trimethylammonium bromide (CTAB) method, and the target gene was amplified by PCR. The primer sequences are listed in the Supplementary Table S1. Finally, the PCR products were sequenced to determine whether gene editing was successful. Only the transformed plants of which the target gene was successfully edited were used in the subsequent experiments.

## Re-sequencing and mutation screening of mutants

For each mutant and wide-type plants, DNA was extracted using the CTAB method. All plant DNA were sequenced on Illumina Hiseq 4000 platform with a coverage depth of ~ 20 × and qualified bases (base quality ≥ 20) over 90% after removing adaptors and low quality reads (i.e. reads contain half of low-quality bases). Cleaned reads were mapped to the reference genome *O. sativa* L. ssp. *japonica* var. Nipponbare (IRGSP-1.0) by BWA-mem (version 0.7.10-r789) with default settings (Li 2013). The resulting bam files were then sorted and processed with MarkDuplicates in Picard package (version 1.114) to remove non-informative PCR duplicates. A local realignment step was also implemented using RealignerTargetCreator and IndelRealigner in GATK package (version 3.5.0.22) to reduce false variant calls due to higher alignment errors around INDELs (insertion/deletion) (DePristo et al. 2011). Variant sites, including SNPs (single nucleotide polymorphisms) and INDELs, were called by Sametools and filtered using custom Python scripts. To check putative off-target effects of CRISPR/Cas9, all the genes of IAA family and ARF family were manually inspected in Intergrative Genomics Viewer (IGV) (Thorvaldsdóttir et al. 2013) to screen out any sequence changes from the wild-type plant. An effective mutation which might influence the phenotype of the mutants was defined when a nucleotide was different from the wild-type sequence(s) and homozygous (the mutated reads > 3, mapping quality ≥ 50 and effective matched degree ≥ 90). Here, “effective matched degree” means the ratio of matched length to whole read length.

### Expression analysis of OsIAA23 in mutant and wild-type rice

Total RNA was prepared from 50 mg tissues from 2-week-old plants or plants in the ripe stage using standard protocols (Takara Code No. 9767). The cDNA samples prepared from no more than 10 µg of total RNA in a total amount of 40 µl was used as template and mixed with a PrimeScript Master Mix and primers for real-time PCR (RT-PCR) analysis (Takara Code No. RR036A), according to the manufacturer’s instructions. Reactions were run in triplicate. The housekeeping gene glyceraldehyde-3-phosphate dehydrogenase (*G3PDH*) was used as an internal control to normalize expression levels. The expression data were analyzed using the 2^(−ΔΔCt)^ method. Each pair of primers designed by Oligo 6 was checked by BLAST in the rice genome sequence to ensure that the amplified sequence was indeed unique. The primer sequences are listed in Supplementary Table S1.

Transcripts from the root tissues of different mutants and wild-type cultivars were PCR amplified using the cDNA library. The PCR DNA fragments were extracted from the agarose gel using a Thermo Scientific GeneJET gel extraction kit. The purified DNA fragments were sequenced in two different ways: by Illumina HiSeq 4000 at a coverage of 200,000 × and by Pacbio Sequel at a coverage of 20,000 ×. The database from Illumina HiSeq 4000, combined 56 published data (Supplementary Table S2), was analyzed using Tophat and Samtools. The database of Pacbio Sequel was mapped in the genome by Gmap and cufflinked by Sametools. The exon–intron junctions and transcripts of different mutants were detected and compared using Python.

## Results

### Evolutionary features of the OsIAA23 gene in the rice genome

**Fig. 1:**
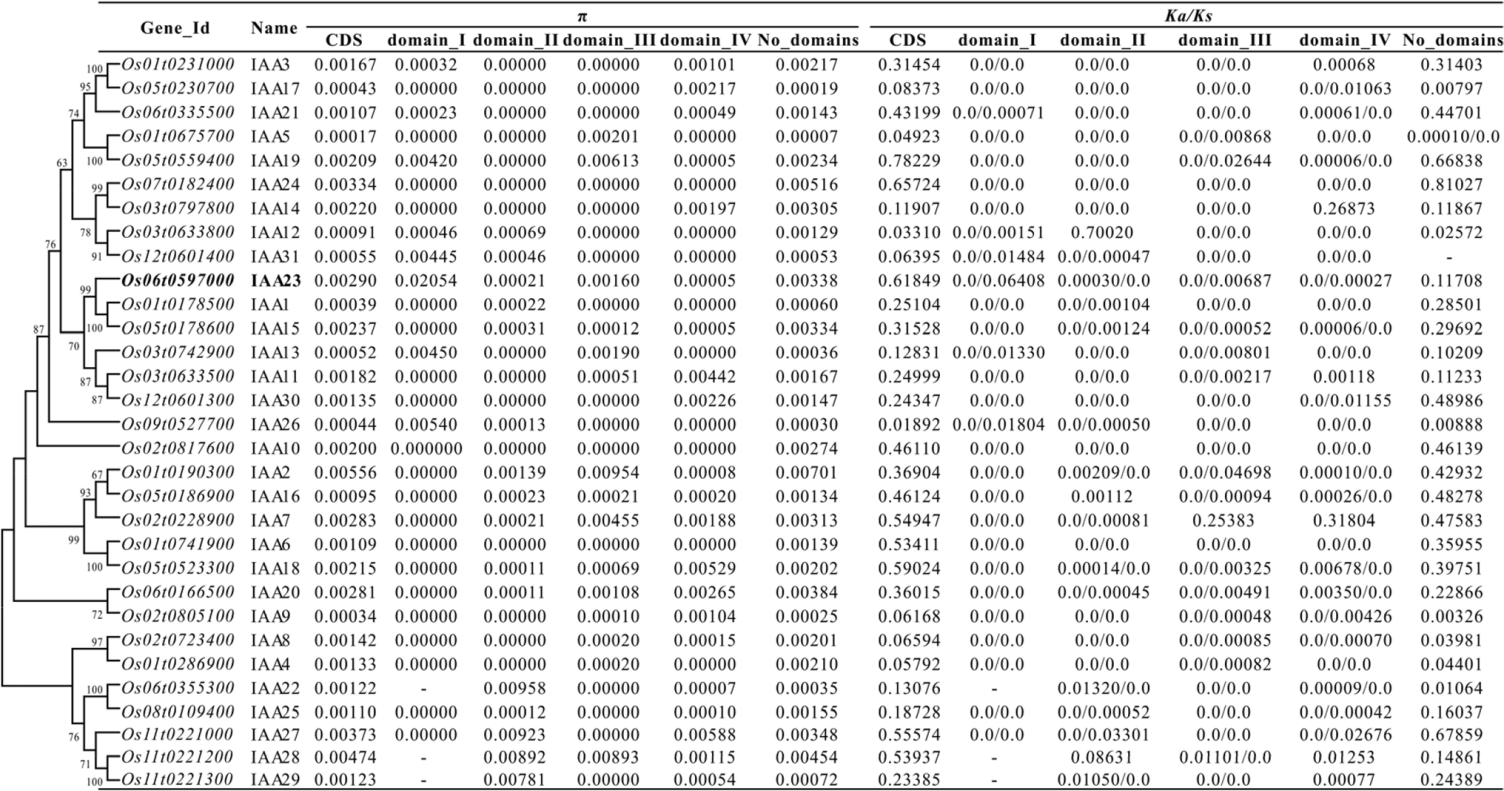
Nucleotide diversity (π) and ratios of nonsynonymous to synonymous nucleotide substitutions (*Ka/Ks*) of *IAA* genes among rice genomes, including 166 *indica* rice, 167 *japonica* rice and 60 wild rice

Thirty-one *IAA* homologs (*IAA1*-*IAA31*) were identified in the rice genome (Mukesh et al. 2006). To estimate the evolutionary features of these *IAA* genes, we calculated nucleotide diversity (π), *Ka/Ks* ratios and Tajima’s D of their CDSs and the main four domains (I–IV) among 393 rice genomes. These rice genomes included 166 *indica*, 167 *japonica*, and 60 wild rice varieties. As Tajima’s D values showed, an excess of low-frequency nucleotide polymorphisms was detected in most *IAA* genes, particularly in Domain II and IV (Supplementary Table S3). The CDS regions of several *IAA* genes showed relatively high polymorphism (≥ 2‰) such as *OsIAA2*, *OsIAA23*, and *OsIAA27* (Fig. 1). For some genes, including *OsIAA2*, *OsIAA22*, *OsIAA23*, and *OsIAA29*, strong selection was detected in their Domain II, which plays a major role in the stability of Aux/IAA proteins.

Phylogenetic reconstruction of the *OsIAAs* (Fig. 1) shows that *OsIAA23* belongs to a small clade, which includes three homologs (*OsIAA1*, *OsIAA15*, and *OsIAA23*). The nucleotide diversity between *OsIAA23* and *OsIAA1*, *OsIAA15* and *OsIAA23*, and *OsIAA1* and *OsIAA15* is 0.293, 0.305, and 0.158, respectively. Compared to its two paralogs, *OsIAA23* exhibits significantly higher nucleotide diversity whether in CDS region, Domain I, or Domain III. Each gene of the clade is old, with each having alleles in other grasses, such as maize, sorghum, and Brachypodium (data not shown), suggesting the conservation and importance of the three genes in the Poaceae.

At the gene expression level, the *OsIAA23* gene and its two paralogs show different tissue expression patterns (Mukesh et al. 2006). *OsIAA23* is highly expressed in the roots, whereas *OsIAA1* and *OsIAA15* are highly expressed in flowers. In addition, unlike *OsIAA1* and *OsIAA15*, the expression of *OsIAA23* is not induced by the auxin analog 2,4-dichlorophenoxyacetic acid (2,4-D). These results suggest that *OsIAA23* may have undergone functional differentiation.

### Targeted mutagenesis of OsIAA23 using the CRISPR/Cas9 system

**Fig. 2:**
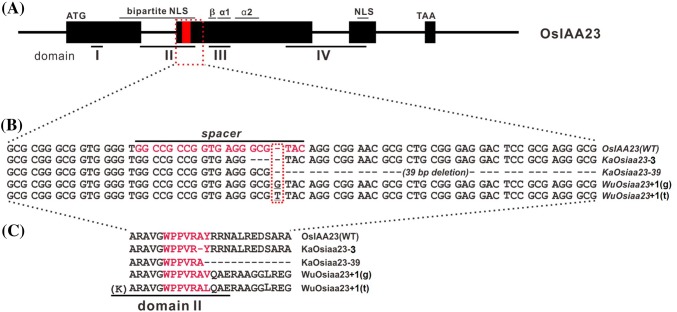
Targeted mutagenesis of *OsIAA23* in rice using CRISPR/Cas system. **a** Diagram of gene structure and conserved domains of *OsIAA23* (Os06t0597000-01 for representation). The black rectangles represent the exon regions. The short lines are marked with Roman numerals bellow indicate four domains of OsIAA23, and the spacer location is marked by red. **b** sgRNA:Cas9-induced rice *OsIAA23* gene mutations at the target site in the mutants. The sequence marked in the red text under the black line indicates the spacer sequence. **c** Amino acid sequence alignment of wild type OsIAA23 protein and the *Osiaa23* mutants. The sequence marked in the red text indicated the spacer location, and Domain II is indicated by underlined text

According to the gene structure and the conserved domains, the specific spacer sequence for *OsIAA23* was designed and is located at the end of Domain II (Fig. 2a, b). Two Kasalath mutants and five Wuyunjing24 mutants were identified by sequencing the target region of *OsIAA23* in the positive transgenic T0 plants (Table 1). Both heterozygous double knockout mutants were found in the two recipient rice varieties (Table 2). Compared to the wild-type Kasalath, all the T0 Kasalath mutants were slightly dwarf, have less tillers, and semi-sterile. However, all the T0 Wuyunjing24 mutants showed no distinguishable mutant phenotype, as indicated by normal tiller number, seed setting rate, and growth cycle, except for slight dwarfing.

**Table 1:** CRISPR/Cas9 editing results of *OsIAA23* in T0 rice plants

Rice varieties	PCR identification	Mutants	Editing rate (%)	Editing type	Mutant genotype
Kasalath	6	2	33.3	double mutant	3 bp-deletion/39 bp-deletion
Wuyunjing24	5	5	100	double mutant	G-insertion/T-insertion

**Table 2:** Genotypes and mutant sequences of T0 plants

T0 lines	Genotype	Sequence	Germination rate (%)
KaOsiaa23-1/3/4/5	wild type	GGGCGTACAGGCGGAACGCGCTGCGGGAGGACGCCGCGAGGGCGAAG	100
KaOsiaa23-2/6	3 bp-deletion/39 bp-deletion	GG-TACAGGCGGAACGCGCTGCGGGAGGACGCCGCGAGGGCGAAG GG-GCGAAG	66.7
WuOsiaa23-1/2/3/4/5	G-insertion/T-insertion	GGGCG **G** TACAGGCGGAACGCGCTGCGGGAGGACGCCGCGAGGGCGAAG GGGCG **T** TACAGGCGGAACGCGCTGCGGGAGGACGCCGCGAGGGCGAAG	60.0

Two mutation types were identified in the Kasalath mutants, namely, a 3-bp deletion that we designated as *KaOsiaa23*-*3*, and a 39-bp deletion named *KaOsiaa23*-*39* (Fig. 2b). Similarly, for the Wuyunjing24 mutants, two mutations were also identified, namely, a G-insertion that we designated as *WuOsiaa23 *+ *1(g)* and a T-insertion we called *WuOsiaa23 *+ *1(t)* (Fig. 2b). In theory, the *KaOsiaa23*-*3* mutant gene encodes a protein that lacks an alanine (Ala, A), whereas the *KaOsiaa23*-*39* protein lacks 13 amino acids (Fig. 2c). However, all the mutations did not affect the core sequence GWPPV of Domain II. For the Wuyunjing24 mutants, both types of single-base insertions are predicted to cause a frame shift in the *OsIAA23* gene, thereby resulting in the deletion of Domains III and IV.

To assess the prevalence of off-target mutations and to further confirm the focal sequence changes, the mutants were re-sequenced and screened for mutation in a genome-wide scale. Firstly, all the genes in the IAA gene family and ARF gene family were investigated to check their mutations in the mutants. Except *OsIAA23*, all the other genes in the Kasalath and Wuyunjing24 mutant genomes showed the same genotype with the corresponding wild-type genome (Supplementary Material 3). We then surveyed the genome-wide variant sites in each mutant in each background. In the three Kasalath mutants, 116 SNPs were called, with 106 in the intergenic region, 5 in the intron region, 3 in the UTR region and 2 in the exon region (Supplementary Figure S1A and Supplementary Material 3). The 2 SNPs located in the two different gene loci, *Os02g0618200* and *Os10g0147400*, resulting a synonymous mutation and a single amino acid change in the non-domain region, respectively. Except the intended deletions due to the role of CRISPR/Cas9, only 4 INDELs were called in the Kasalath mutants, including 3 in the intergenic region and 1 in the intron region. In the two Wuyunjing24 mutants, *WuOsiaa23 *+ *1(g)* and *WuOsiaa23 *+ *1(t)*, 61 SNPs and 12 INDELs were totally identified, except the G/T-insertion (Supplementary Figure S1B and Supplementary Material 3). Only 2 SNPs located in the exon region while all the other SNPs and INDELs were found in the non-coding regions. The 2 SNPs also resided in two different gene loci, *Os06g0129000* and *Os11g0670700*, both encoding hypothetical proteins, without function reports.

### Phenotypic assessment of *Osiaa23* mutant

The T1 generation seeds of the heterozygous mutants of Kasalath and Wuyunjing24 were grown in Murashige and Skoog (MS) basal medium for 1 days under the same conditions for observation and statistical analysis. For Kasalath, of the 20 mutant plants, 10 were heterozygous *KaOsiaa23*-*3/*-*39* mutants, four were homozygous *KaOsiaa23*-*3* mutants, and the remaining six were homozygous *KaOsiaa23*-*39* mutants (Supplementary Table S4). For Wuyunjing24, of the 30 mutant plants, 15 were heterozygous *WuOsiaa23 *+ *1(g)/*+ *1(t)* mutants, eight were homozygous *WuOsiaa23 *+ *1(g)* mutants, and the remaining seven were homozygous *WuOsiaa23 *+ *1(t)* mutants (Supplementary Table S4).

**Fig. 3:**
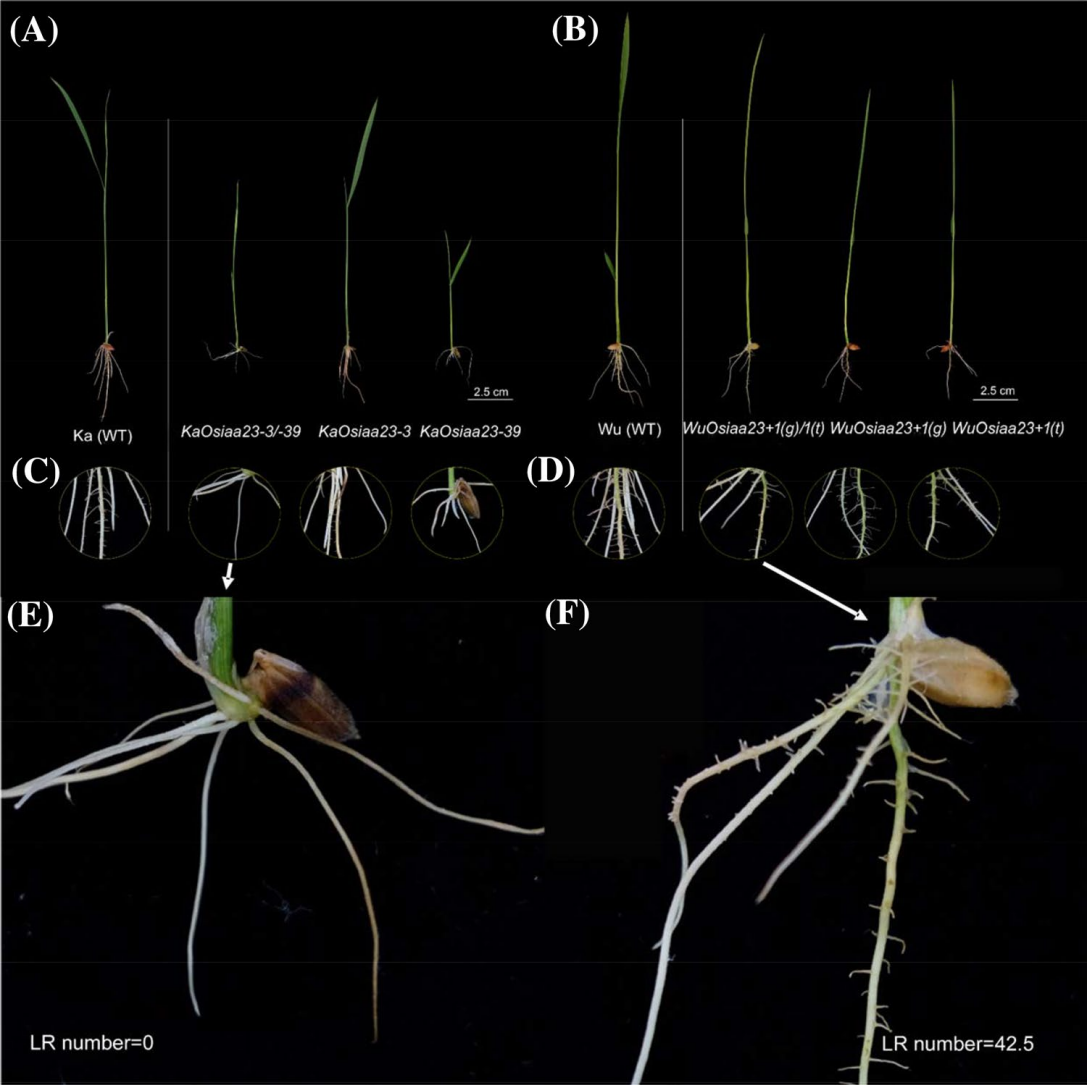
Phenotypes of 7-day-old seedlings of wild type plants (WT), mutants of *Osiaa23*. Phenotype of 7-day-old seedlings of wild-type and mutants of Kasalath (**a**) and Wuyunjing24 (**b**), Bar = 2.5 cm. As marked, from left to right: wild-type (WT), the heterozygous mutants, and the homozygous mutants. Root phenotype of 7-day-old seedlings of wild-type and mutants of Kasalath (**c**) and Wuyunjing24 (**d**), Diameter = 1.4 cm. Enlarge views of root of the heterozygous mutants of Kasalath (**e**) and Wuyunjing24 (**f**)

**Fig. 4:**
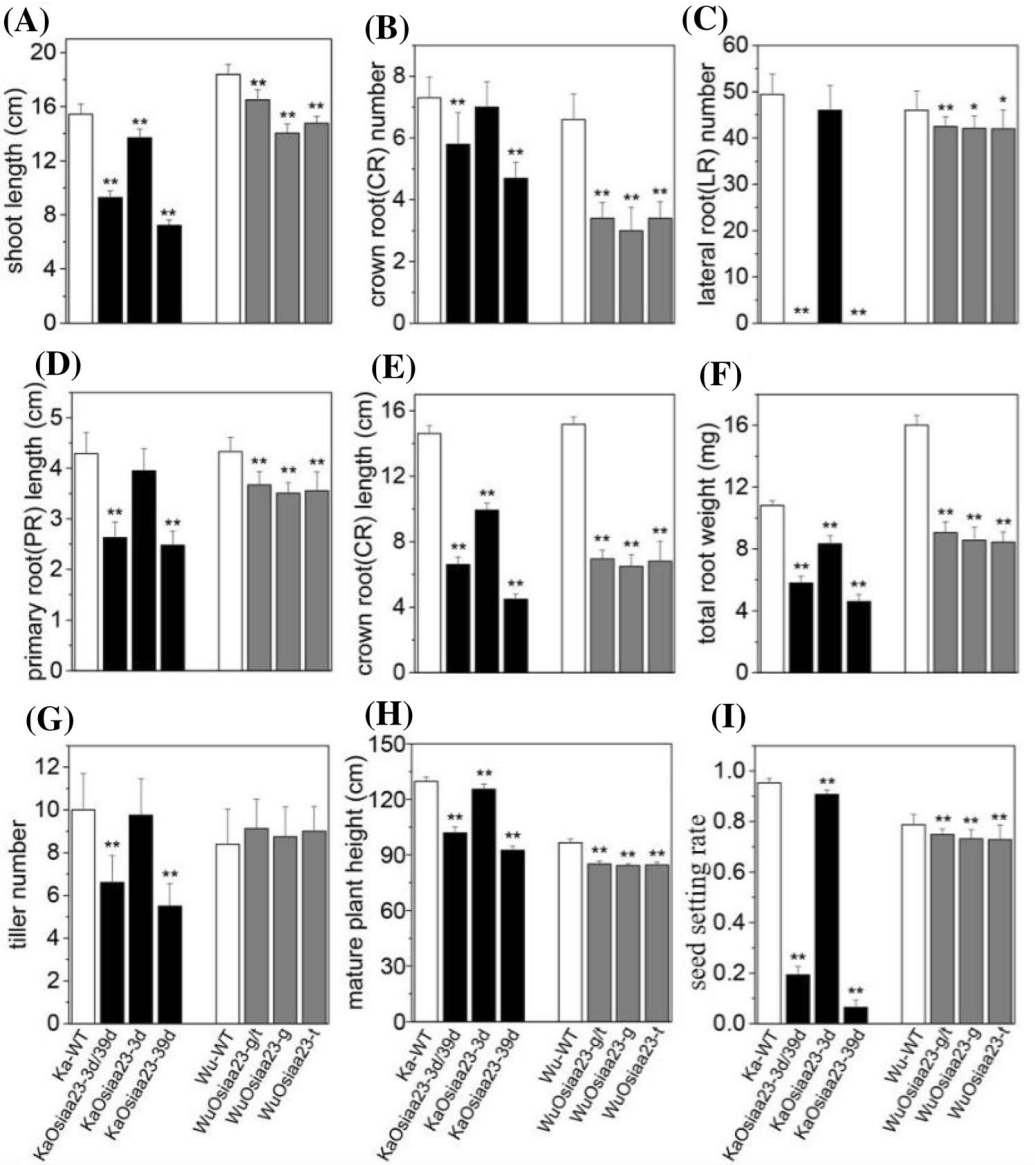
Growth parameters of wild type plants (WT), mutants of *Osiaa23*. The phenotypes of *Osiaa23* mutants are compared with WT in the aspects of shoot length (**a**), crown root number (**b**), lateral root number (**c**), primary root length (**d**), crown root length (**e**), total root (fresh) weight (**f**), tiller number (**g**), mature plant height (**h**) and seed setting rate (**i**). Black columns represent Kasalath mutants, and gray columns represent Wuyunjing24 mutants. As marked at the bottom, from left to right: wild-type plants (WT), the heterozygous mutants, and the homozygous mutants. The standard deviation of the mean was denoted as error bar for each column. *p < 0.05, **p < 0.01 (Student’s *t* test)

In general, all the mutants in the seedling stage showed similar mutant phenotypes in height, crown root, primary root, and lateral root (Fig. 3). First, we found that all the mutants were of smaller stature than the two wild-types (Figs. 3a, b, 4a and Supplementary Table S4). Among these, mutants *KaOsiaa23*-*3/*-*39* and *KaOsiaa23*-*39* exhibited severe dwarfism (≤ 10 cm), significantly shorter than Kasalath. Second, except for mutant *KaOsiaa23*-*3*, all the other mutants produced significantly fewer crown roots. Meanwhile, the length of the crown roots of all the mutants was significantly shorter than for the two wild-types (Figs. 3a–d, 4b and Supplementary Table S4). Also known as adventitious roots, crown roots are the main roots of rice plants, and a reduction in their number and length reduces seedling height. Third, mutants *KaOsiaa*-*3/*-*39* and *KaOsiaa*-*39* showed extreme inhibition of lateral root formation. Another Kasalath mutant, *KaOsiaa*-*3*, showed slightly fewer lateral roots than the wild-type Kasalath. Similarly, all the Wuyunjing24 mutants also showed slight inhibition of lateral root elongation, consistence with the reduced number of crown roots (Figs. 3a–d, 4c and Supplementary Table S4). This agrees with the previously reported phenotype of *Osiaa23* homozygous mutants (Ni et al. 2011). Lateral roots play an important role in rice; these are responsible for nutrient and water uptake. The inhibition of lateral root formation and elongation might be responsible for the observed abnormal mutant phenotype. Finally, the primary root length was similar to that of the shoot and crown root lengths, although no significant reduction was detected in the mutant *KaOsiaa*-*3*. Similarly, the fresh weight of the total roots of all six mutants was significantly lower than for the two wild-types (Fig. 4d–f and Supplementary Table S4).

**Fig. 5:**
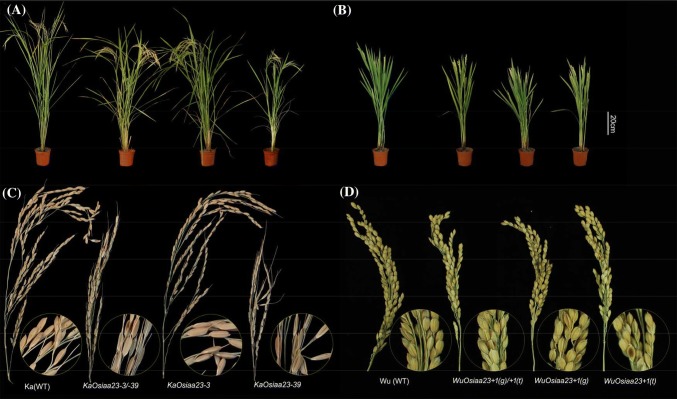
Phenotypes of mature plants of wild type (WT) and mutants of *Osiaa23*. Phenotype of plants at full ripening stage of wild-type (WT) and mutants of Kasalath (**a**) and Wuyunjing24 (**b**), Bar = 20 cm. Phenotypes of spikelet of wild-type (WT), mutants of Kasalath (**c**) and Wuyunjing24 (**d**). As marked at the bottom, from left to right: wild-type (WT), the heterozygous mutants, and the homozygous mutants

Seven-day-old seedlings were transplanted into planting boxes and placed outdoors. Wild-type plants and mutants of Kasalath and Wuyunjing24 were planted in the same box. After about 125 days and 145 days, all the wild-type plants and mutants of Kasalath and Wuyunjing24 reached the full ripening stage, respectively (Supplementary Table S5). Compared to the wild-type, the number of tillers of the two mutants, *KaOsiaa23*-*3/*-*39* and *KaOsiaa23*-*39*, was significantly reduced, whereas the other mutants showed no special phenotype in tiller number (Fig. 5 and Supplementary Table S5). However, the height of all mature plant mutants was significantly lower than that of the two wild-types (Fig. 5a, b and Supplementary Table S5). Especially for the two mutants carrying the 39-bp deletion, these were about 30 cm shorter than the wild Kasalath. Another important and distinct mutant phenotype is their seed setting rate. All the mutants had a significantly lower seed setting rate compared to the wild-type, indicating that the *OsIAA23* gene indirectly affects rice production. However, the production of the mutants also significantly varied. The mutant *KaOsiaa23*-*3* had the same ear type as the wild Kasalath, and all the seeds are plump and full seeds (Fig. 5c). In contrast, the spikelets of the two mutants carrying the 39-bp deletion had no pedicels and the stalk supporting the spikelet on the panicle branch, and most seeds were empty and blighted, thereby resulting in an extremely low seed setting rate.

### Quantification of *OsIAA23* mRNA expression

**Fig. 6:**
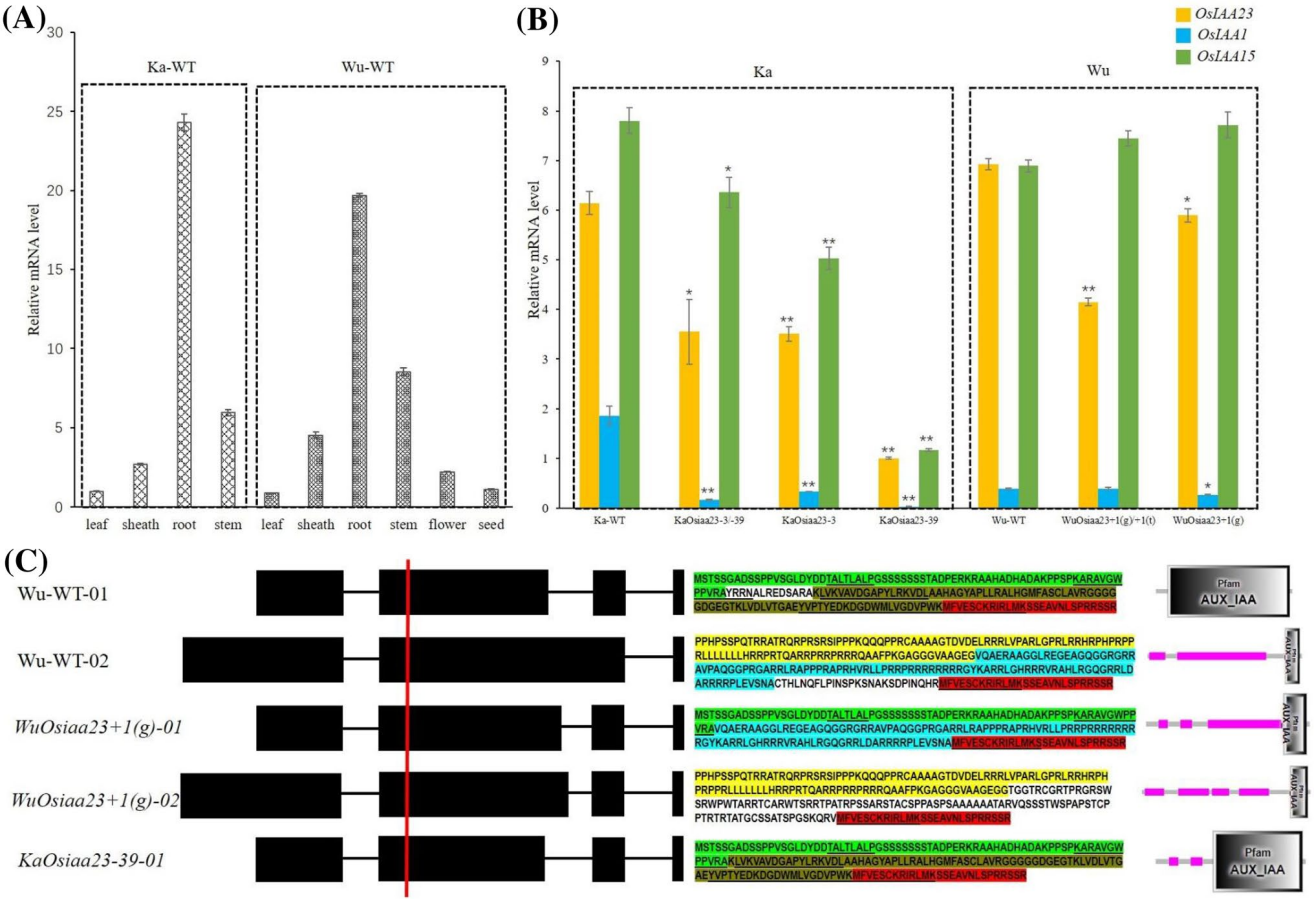
Expression models of *OsIAA23* and its homologs. Real-time PCR expression profiles of *OsIAA23* gene in different tissues of wild-type (Ka-WT) and Wuyunjing24 (Wu-WT) (**a**). Comparison of expression levels of *OsIAA23*, *OsIAA1* and *OsIAA15* in the root of different mutants (**b**). The predicted transcripts, protein sequences and structures of *OsIAA23* gene and edited *Osiaa23* gene (**c**). The red vertical line marks the position of the gene editing. The amino acid sequences highlighted with the same color are the same. The sequences of Domain I, II, III and IV are indicated orderly by underline text. The standard deviation of the mean was denoted as error bar for each column. *p < 0.05, **p < 0.01

The *OsIAA23* gene had the similar expression profile in the wild-type Kasalath and Wuyunjing24, exhibiting the highest expression levels in the roots, followed by the stem, and showed less expression in the other tissues such as the sheaths, leaves, flowers, and seeds (Fig. 6a). This expression pattern coincides with the phenotype of the *Osiaa23* mutants that we observed, which had defects involving initiation of crown roots and lateral roots, and with respect to overall height.

To test whether the two homologs of *OsIAA23*, *OsIAA1* and *OsIAA15,* could compensate the function of destroyed *Osiaa23* in the mutants, RT-PCR analysis of the roots of the mutants and its wild-type plants was performed to assess their expression levels (Fig. 6b). The three homologs exhibited similar expression profiles between wild-type Kasalath and Wuyunjing24, i.e., *OsIAA23* and *OsIAA15* had a higher expression levels, whereas *OsIAA1* had a relatively lower expression level. For the Kasalath mutants, all showed a reduction in expression of edited *OsIAA23*, particularly the homozygous mutants *KaOsiaa23*-*39*. It seems that lower *Osiaa23* expression leads to a more striking mutant phenotype in the Kasalath variety. Interestingly, the other two homologs, particularly *OsIAA15*, show a similar downregulated expression pattern as that of *Osiaa23*, suggesting that the two homologs do not compensate for the function of *Osiaa23*, but that the three genes seem to cooperate together in a co-expression pattern. The expression of *Osiaa23* was slightly decreased in the homolog mutant and further decreased in the heterozygous mutant, whereas the expression of *OsIAA1* and *OsIAA15* was not reduced in the mutants. The mutation type in Kasalath is a three-nucleotide deletion, whereas a single base-pair insertion, which may result in frameshift, was found in Wuyunjing24. Then, we expect that the phenotypic change in Wuyunjing24 mutants should be more obvious and significant. Meanwhile, the transcripts of the Wuyunjing24 *Osiaa23* mutant might also be degraded because of premature transcription termination. However, a slightly mutant phenotype and high expression levels of the mutated *Osiaa23* were observed in the Wuyunjing24 mutants. Is it possible that the mutated *Osiaa23* gene in Wuyunjing24 underwent an alteration in transcription to avoid premature termination?

To assess this question, we examined the genome file of *O. sativa* (IRGSP 1.0), which contains two annotated transcripts (Os06t0597000-01 and Os06t0597000-02), encoding proteins of 193 and 262 amino acids, respectively. Only the last 27 amino acids are identical in the two predicted proteins. We interrogated public RNA-Seq data of rice to determine whether the *OsIAA23* gene has other transcripts. Interestingly, we found several new exon–intron junctions (Supplementary Figure S1). Two of the new junctions are respectively 11-bp (chr06:23502684-23502763) and 13-bp (chr06:23502686-23502763) distal to the annotated second intron of the transcript Os06t0597000-01. If a single base-pair insertion was induced in the second exon before the second intron, just as we found in the edited *Osiaa 23* in Wuyunjing24 mutants, then the gene could adopt a new transcription pattern with the new junction producing a functional mRNA. For Os06t0597000-01, the new predicted mRNA would translate a novel mosaic protein, with the former 71 amino acids similar to the protein translated by Os06t0597000-01 and the latter 127 amino acids the same with the protein translated by Os06t0597000-02 (Fig. 6c). For Os06t0597000-02, on the other hand, the predicted mRNA would translate a new auxin/IAA protein, as indicated in Fig. 6c. By analyzing RNA-seq produced by Illumina Hiseq 4000 in wild-type Wuyunjing24 and mutant plants, we were indeed able to detect these two new junctions (chr06:23502684-23502763 and chr06:23502686-23502763) (Supplementary Table S6). Although the vast majority of the exon–intron junctions in the wild type and mutants are still the annotated ones (99%), the proportion of the two new junctions in WuOsiaa23 mutants increased by 2 to ninefold relative to the wild Wuyunjing24 control. Similarly, the junction chr06:23502686-23502763 was also found in Kasalath and *KaOsiaa23* mutants while the junction chr06:23502684-23502763 was only observed in the wild Kasalath control (Supplementary Table S7). The Pacbio data of RNA-seq was successful in resolving the puzzle by providing the complete sequences of the new transcript isoforms predicted in Fig. 6c.

## Discussion

### Pleiotropic effects of OsIAA23 gene on rice and its role in grass

Traditionally, plant mutants have been generated by ethyl methanesulfonate (EMS), irradiation, T-DNAs or transposons. These mutagenetic methods are usually random and often labor-intensive and time-consuming for identification the causal mutation. The CRISPR/Cas9 gene editing technology could provide precise and highly efficient modifications in specific regions. It also allows rapid identification of causal mutations. Here, we used the CRISPR/Cas9 system and isolated several *Osiaa23* mutants in *japonica* (Wuyunjing24) and *indica* (Kasalath) rice. Both heterozygous double knockout mutants were obtained, with deletions in Kasalath and insertions in Wuyunjing24, indicating the high efficiency and diversity of the editing function of the CRISPR/Cas9 system.

The previously reported EMS-mutagenized *Osiaa23* mutant, which is generated by a point mutation in the core sequence of Domain II, exhibits pleiotropic defects in its root tissues, including crown roots, lateral roots, and root cap (Ni et al. 2011). In the former study, the heterozygous mutants were intermediate in phenotype and exhibited normal fertility, whereas the homozygous mutant showed loss of lateral and crown root primordia, the root gravitropic response and abnormal fertility. An amino acid substitution (G to E) in the conserved core sequence of Domain II of the OsIAA23 protein could cause pleiotropic defects in growth and development, suggesting the importance of the core sequence GWPPV. Here, we generated several mutants using mutations just following the core sequence GWPPV. In the Kasalath mutants, three-nucleotide and 39-nucleotides deletions were detected, resulting in an alanine deletion and a 13-amino acid deletion, respectively. Mutants *KaOsiaa23*-*3/*-*39* and *KaOsiaa23*-*39*, both carrying the 39-bp deletion, showed the most obvious and significant mutant phenotypes, especially the homozygous mutant. Defects in the root tissues of mutants then result in extremely low fertility rates. In contrast, the deletion of the alanine residue after the core sequence of the Domain II in mutant *KaOsiaa23*-*3* mainly resulted in limited crown root length. This indicates that the absence of these amino acids affects the normal metabolism of the OsIAA23 protein, but the degree of reduction was weaker than the previously reported *Osiaa23*, suggesting the importance of the core sequence GWPPV of Domain II.

The root system of monocots and dicots differs in terms of architecture (Smith and De Smet 2012). Most dicots have a primary root and several lateral roots. The grasses often have a fibrous root system and the branching roots are produced through crown roots. Crown roots are much more vital for the root architecture of grasses than in dicots. Auxin induces lateral-root formation in dicots and crown root formation in grasses (McSteen 2010). It appears that crown root initiation is similarly controlled by auxin to initiate lateral root formation. However, crown roots are developed from shoots, whereas lateral roots are produced from root tissues. There thus might be specific genes that are regulated by auxin that control crown root growth in grasses. Here, no *OsIAA23* homolog (amino acids similarity > 30%) was identified in *A. thaliana*, and the *OsIAA23* gene is specific to grass. One of the main functions of *OsIAA23* is related to crown roots or adventitious roots, which is grass-specific. It is possible that the grass-specific *OsIAA23* gene was generated after the appearance of the grasses for crown root initiation. About seven *OsIAA* genes in rice have been reported, only *OsIAA23* was found to have the effect on the crown roots formation (Nakamura et al. 2006; Song et al. 2009; Ni et al. 2011, 2014; Zhu et al. 2012; Kitomi et al. 2012; Song and Xu 2013). Thus, the grass-specific *OsIAA23* gene might play one of the major roles for root architecture in grasses.

## Interactions among *OsIAA23* and its two homologs

Aux/IAA proteins are usually characterized by four highly conserved domains I–IV (Mukesh et al. 2006). Domain I functions as an active repression domain, and Domain II is responsible for the protein Aux/IAA stability. Some Aux/IAA proteins lack an apparent Domain II, and some lack both Domains I and II and do not function as repressors in the auxin signaling pathway (Tiwari et al. 2004; Dreher et al. 2006). Domains III and IV are protein–protein interaction domains, facilitating the formation of ARF–ARF, ARF-Aux/IAA and Aux/IAA- Aux/IAA homo- and heterodimers. As auxin response factors, Aux/IAA proteins usually form multimers with ARFs. Recently, many studies on interactions between Aux/IAA and Aux/IAA proteins have been reported. Based on large-scale interactome analyses, such as yeast two-hybrid, bimolecular fluorescence complementation (BiFC) experiments, a comprehensive physical interactome map of Aux/IAA proteins has been developed in *Arabidopsis thaliana*, indicating Aux/IAA-Aux/IAA interactions are common (Vernoux et al. 2011; Luo et al. 2018). In maize, ZmIAA15 was found to have interaction both with ZmIAA10 and ZmIAA29. And ZmIAA23 also interacted with ZmIAA10 as well as ZmIAA29 (Ludwig et al. 2014).

Here, only Domain II was damaged in *Osiaa23* in Kasalath mutants, and Domains I, III, and IV remained intact. The expression of *Osiaa23* sharply decreased, particularly in the homozygote mutant *KaOsiaa23*-*39*, which harbored a 13-amino acid deletion downstream of core of Domain II. With the decrease in the expression level of *Osiaa23*, its two homologs, *OsIAA1* and *OsIAA15*, also showed reduced expression. It seems that in the Kasalath mutants, *Osiaa23* and its two homologs cooperate or are involved in the same pathway such as auxin signaling. The three proteins might form hetero-oligomers as they all have the intact interaction domains III and IV. Their interactions result in the observed mutant phenotype. In Wuyunjing24 mutants, although the expression level of *Osiaa23* slightly decreased, the expression of *OsIAA1* and *OsIAA15* did not significantly decrease. This might be because the edited *Osiaa23* has recovered its partial function, and its partners function normally. Unlike the Kasalath mutants, the Wuyunjing24 mutants harbor a frameshift mutation in *Osiaa23*, which might be eliminated by alternative splicing, resulting in a weak mutant phenotype. Based on the RT-PCR results, we predict that *OsIAA23* might cooperate with its homologs, particularly *OsIAA15*. When the function of *OsIAA23* is destroyed, the two homologs also reduce their expression; when the function of *OsIAA23* is recovered, the two homologs retain normal expression level. More experiments are needed to test their relationships. It is possible that homodimers or heterodimers of Aux/IAA proteins might be involved with the regulation of auxin response genes. Then the Aux/IAA homo- or hetero-dimers form higher order multimers with ARF proteins and block the transcriptional activation of auxin-responsive genes.

## Electronic supplementary material

Below is the link to the electronic supplementary material.

Supplementary material 1 (PDF 191 kb)

Supplementary material 2 (DOCX 32 kb)

Supplementary material 3 (XLSX 20 kb)
