# Supplementary material for: Different knockout genotypes of OsIAA23 in rice using CRISPR/Cas9 generating different phenotypes
Source: Plant Mol Biol. 2019 Apr 19;100(4):467–79. doi: 10.1007/s11103-019-00871-5 (PMC6586719; doi:10.1007/s11103-019-00871-5)

Fig.S1 The mutation screening results of the Kasalath (A) and Wuyung24 (B) mutants.

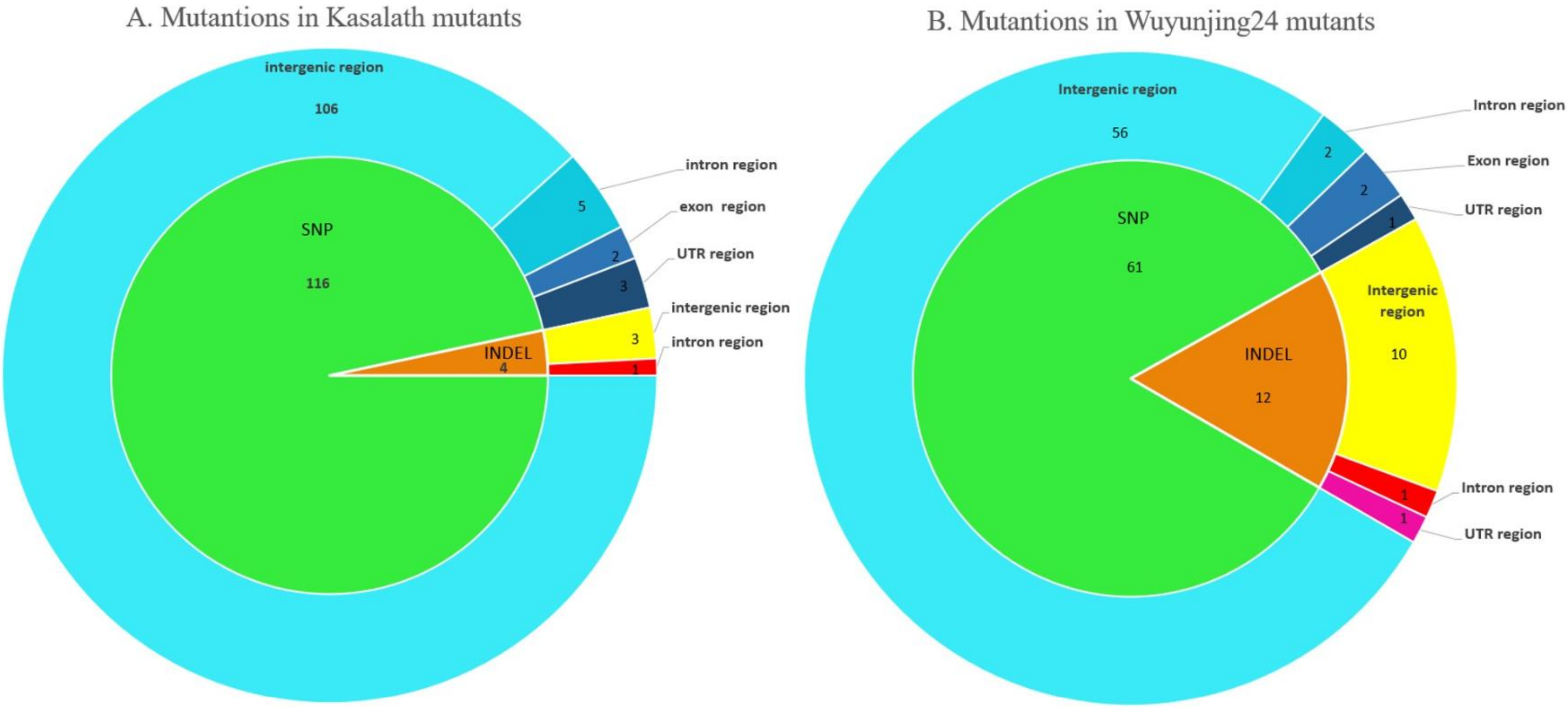

Fig.S2 Diagram of splice junctions of OsIAA23 predicted from the genome file of O. sativa (IRGSP1.0).

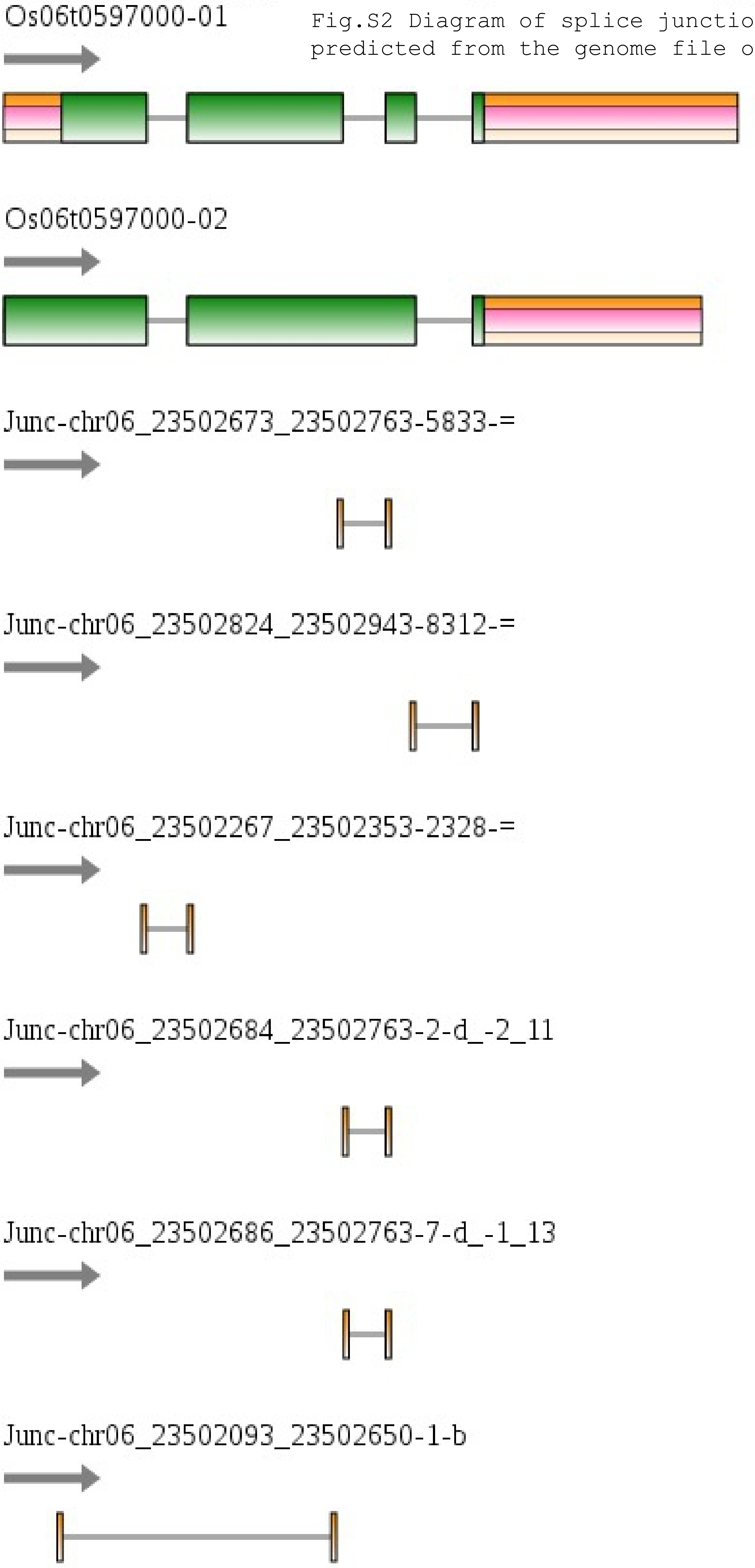

Supplement: Supplementary file 1 — Supplementary material 1 (PDF 191 kb) [file 11103_2019_871_MOESM1_ESM.pdf]
