# Supplementary material for: Different knockout genotypes of OsIAA23 in rice using CRISPR/Cas9 generating different phenotypes
Source: Plant Mol Biol. 2019 Apr 19;100(4):467–79. doi: 10.1007/s11103-019-00871-5 (PMC6586719; doi:10.1007/s11103-019-00871-5)
Supplement: Supplementary file 2 — Supplementary material 2 (DOCX 32 kb) [file 11103_2019_871_MOESM2_ESM.docx]

**Different knockout genotypes of *OsIAA23* in rice using CRISPR/Cas9 generating different phenotypes**

Mengmeng Jiang^1^, Huaying Hu^1^, Jing Kai, Milton Brian Traw, Sihai Yang^2^, Xiaohui Zhang^2^

State Key Laboratory of Pharmaceutical Biotechnology, School of Life Sciences, Nanjing University, 210023, Nanjing, China

^1^ M.J. and H.H. contributed equally to this work.

^2^ To whom correspondence should be addressed.

Tel: +86-25-89686406.

Email addresses: sihaiyang@nju.edu.cn (Sihai Yang),

xiaohuizhang@nju.edu.cn (Xiaohui Zhang).

**Table S1.** The primer sequences used in this study.

| Primer name | Sequences (5’ to 3’) |
| --- | --- |
| Spacer-U | GGCAGGCCGCCGGTGAGGGCGTAC |
| Spacer-L | AAACGTACGCCCTCACCGGCGGCC |
| Crispr-pcr-U | GACTACGACGACACCGCCCT |
| Crispr-pcr-L | CCGGTGACCAAGTCGACGAG |
| OsIAA23-Qpcr-U | TGCCCACCTACGAGG AC |
| OsIAA23-Qpcr-L | CGTCTTGGCGATAAGTTGA |
| OsIAA1-Qpcr-U | CTGTG CTGGCAATCGTG |
| OsIAA1-Qpcr-L | CGTAGCCGACGTGGATGT |
| OsIAA15-Qpcr-U | AAGGCCAAGTT GTGAAG |
| OsIAA15-Qpcr-L | GTCGGCACGTACTCGTTG |
| OsIAA23-transcript -A2U | CCACACCCCTCGTCTCC |
| OsIAA23-transcript -AlU | CTCGGGCCTCGACTACG |
| OsIAA23-transcript -AlL | TATCTGGATGATCGTCTTGG |
| G3DPH-U | CATCATTCCCAGCAGCAC |
| G3DPH-L | GCAGCCTTCTCGATTCTAAC |

Table S2. The published database used in this study.

| **No.** | **Database** | **Data(G)** | **Read length** | **Merged(Y/N)** | **Progress** | **Sra**^†^ |
| --- | --- | --- | --- | --- | --- | --- |
| DB001 | 9311_Seedling_Shoots_GR-Lu2010 | 6.07 | 40~76 | Y | Bam,Cufflink | ERR008647 ERR008648 ERR008653 ERR008654 ERR008659 ERR008660 |
| DB002 | Guangluai4_Seedling_Shoots_GR-Lu2010 | 6.14 | 40~76 | Y | Bam,Cufflink | ERR008649 ERR008650 ERR008656 ERR008661 ERR008662 |
| DB003 | Nipponbare_Seedling_Shoots_GR-Lu2010 | 6.09 | 40~76 | Y | Bam,Cufflink | ERR008651 ERR008652 ERR008657 ERR008658 ERR008664 |
| DB004 | Nipponbare_Flowering_Leaf_RNA-Lu2015 | 55.8 | 150 | N | Bam,Cufflink | ERR855945 |
| DB005 | Nipponbare_Flowering_Panicle_RNA-Lu2015 | 58.6 | 150 | N | Bam,Cufflink | ERR855947 |
| DB006 | 9311_Callus_GR-Zhang2010 | 14.03 | 35~75 | Y | Bam,Cufflink | SRR037711 SRR037712 SRR037713 SRR037714 SRR037715 SRR037716 SRR037717 SRR037718 SRR037719 SRR037720 SRR037721 SRR037722 SRR037723 SRR037724 |
| DB007 | 9311_Booting_Panicle_GR-Zhang2010 | 13.58 | 35~75 | Y | Bam,Cufflink | SRR037725 SRR037726 SRR037727 SRR037728 SRR037729 SRR037730 SRR037731 SRR037732 SRR037733 SRR037734 SRR037735 SRR037736 SRR037737 SRR037738 SRR037739 |
| DB008 | 9311_Seedling_Shoots_GR-Zhang2010 | 0.1360807 | 35 | N | Bam | SRR037740 |
| DB009 | 9311_Seedling_Root_GR-Zhang2010 | 0.180312685 | 35 | N | Bam | SRR037741 |
| DB010 | 9311_Tillering_Leaf_GR-Zhang2010 | 0.20481083 | 35 | N | Bam | SRR037742 |
| DB011 | 9311_Flowering_Panicle_GR-Zhang2010 | 0.185004015 | 35 | N | Bam | SRR037743 |
| DB012 | 9311_Flowering_Leaf_GR-Zhang2010 | 0.190336825 | 35 | N | Bam | SRR037744 |
| DB013 | 9311_Filling_Panicle_GR-Zhang2010 | 0.197727775 | 35 | N | Bam | SRR037745 |
| DB015 | Pa64s_BGI | 14.16 | 49 | Y | Bam,Cufflink | SRR923813 SRR923816 SRR923817 |
| DB016 | N2Y6_Filling-E_Leaf | 0.6 | 36 | N | Bam,Cufflink | SRR1609318 |
| DB017 | N2Y6_Filling-M_Leaf | 0.26 | 36 | N | Bam,Cufflink | SRR1609319 |
| DB018 | N2Y6_Filling-L_Leaf | 0.42 | 36 | N | Bam,Cufflink | SRR1609320 |
| DB019 | LYP9_Filling-E_Leaf | 0.62 | 36 | N | Bam,Cufflink | SRR1609321 |
| DB020 | LYP9_Filling-M_Leaf | 0.37 | 36 | N | Bam,Cufflink | SRR1609322 |
| DB021 | LYP9_Filling-L_Leaf | 0.39 | 36 | N | Bam,Cufflink | SRR1609323 |
| DB022 | W1921_Seedling_Shoots | 11.1 | 101 | N | Bam,Cufflink | DRR001371 |
| DB023 | Kasalath_Seedling_Shoots | 10.41 | 101 | N | Bam,Cufflink | DRR001372 |
| DB024 | NipponbarexKasalath_Seedling_Shoots | 10.1 | 101 | N | Bam,Cufflink | DRR001373 |
| DB025 | W0106_Seedling_Shoots | 14.7 | 101 | N | Bam,Cufflink | DRR001374 |
| DB026 | KasalathxNipponbare_Seedling_Shoots | 13.2 | 101 | N | Bam,Cufflink | DRR001375 |
| DB027 | Oryza-glaberrima_leaf | 43.3 | 100 | N | Bam,Cufflink | SRR1174376 |
| DB028 | Oryza-glaberrima_panicle | 25.45 | 100 | N | Bam,Cufflink | SRR1174378 |
| DB029 | Oryza-glaberrima_root | 37.55 | 100 | N | Bam,Cufflink | SRR1174379 |
| DB030 | Nipponbare_Flowering-E_Buds_TPJ-Wang2015 | 53.4 | 101 | N | Bam,Cufflink | SRR1213581 |
| DB031 | Nipponbare_Flowering_Flower_TPJ-Wang2015 | 56.11 | 101 | N | Bam,Cufflink | SRR1213582 |
| DB032 | Nipponbare_Flowering-E_Leaf_TPJ-Wang2015 | 58.53 | 101 | N | Bam,Cufflink | SRR1213583 |
| DB033 | Nipponbare_Flowering-L_Leaf_TPJ-Wang2015 | 56.58 | 101 | N | Bam,Cufflink | SRR1213584 |
| DB034 | Nipponbare_Flowering-E_Root_TPJ-Wang2015 | 52.57 | 101 | N | Bam,Cufflink | SRR1213585 |
| DB035 | Nipponbare_Flowering-L_Root_TPJ-Wang2015 | 58.17 | 101 | N | Bam,Cufflink | SRR1213586 |
| DB036 | Nipponbare_Filling_Grains_TPJ-Wang2015 | 52.26 | 101 | N | Bam,Cufflink | SRR1213587 |
| DB037 | Nipponbare_Ripening_Seed_TPJ-Wang2015 | 58.42 | 101 | N | Bam,Cufflink | SRR1213588 |
| DB038 | Oryza-meridionalis_Panicle | 50.4 | 100 | N | Bam,Cufflink | SRR1174791 |
| DB039 | Oryza-meridionalis_Root | 49.53 | 100 | N | Bam,Cufflink | SRR1174792 |
| DB040 | Oryza-meridionalis_leaf | 51.61 | 100 | N | Bam,Cufflink | SRR1174790 |
| DB041 | TP309_Seedling_Leaf | 19.1 | 100 | N | Bam,Cufflink | ERR194168 |
| DB042 | Oryza-barthii_Leaf | 9.63 | 120 | N | Bam,Cufflink | SRR1170742 |
| DB043 | Oryza-barthii_Root | 9.12 | 120 | N | Bam,Cufflink | SRR1170744 |
| DB044 | Oryza-barthii_Panicle | 48.64 | 100 | N | Bam,Cufflink | SRR1170762 |
| DB045 | Nipponbare_Callus_0DAC | 22.21 | 150 | N | Bam,Cufflink | SRR4241116 |
| DB046 | Nipponbare_Callus_2DAC | 21.51 | 150 | N | Bam,Cufflink | SRR4241117 |
| DB047 | Nipponbare_Callus_5DAC | 20.69 | 150 | N | Bam,Cufflink | SRR4241118 |
| DB048 | Nipponbare_Seedling_Root_Rep01 | 5.43 | 100 | N | Bam,Cufflink | SRR1616169 |
| DB049 | Nipponbare_Seedling_Root_Rep02 | 5.16 | 100 | N | Bam,Cufflink | SRR1616570 |
| DB050 | Nipponbare_Seedling_Root_Rep03 | 3.56 | 100 | N | Bam,Cufflink | SRR1616571 |
| DB051 | Nipponbare_Seedling_Shoots_Rep01 | 3.76 | 100 | N | Bam,Cufflink | SRR1616572 |
| DB052 | Nipponbare_Seedling_Shoots_Rep02 | 4.01 | 100 | N | Bam,Cufflink | SRR1616573 |
| DB053 | Nipponbare_Seedling_Shoots_Rep03 | 3.85 | 100 | N | Bam,Cufflink | SRR1616574 |
| DB054 | Oryza-rufipogon_Leaf | 41.8 | 100 | N | Bam,Cufflink | SRR1220645 |
| DB055 | Oryza-rufipogon_Panicle | 38.4 | 100 | N | Bam,Cufflink | SRR1220646 |
| DB056 | Oryza-rufipogon_Root | 19.6 | 100 | N | Bam,Cufflink | SRR1220647 |

^†^ Sra: Sequence Read Archive.

**Table S3.** Tajima's D of *IAA* genes among rice genomes.

| Name | Gene_Id | cds | domain Ⅰ | domain_II | domain_III | domain_IV | No_domains |
| --- | --- | --- | --- | --- | --- | --- | --- |
| OsIAA1 | Os01t0178500 | -1.754 | n/c | -1.196 | n/c | n/c | -1.558 |
| OsIAA2 | Os01t0190300 | 0.297 | n/c | -0.977 | 1.854 | -0.877 | 0.421 |
| OsIAA3 | Os01t0231000 | -1.217 | -0.875 | n/c | n/c | -0.836 | -1.033 |
| OsIAA4 | Os01t0286900 | -0.651 | n/c | n/c | -0.851 | n/c | -0.455 |
| OsIAA5 | Os01t0675700 | -1.707 | n/c | n/c | -0.37 | n/c | -1.771 |
| OsIAA6 | Os01t0741900 | -0.993 | n/c | n/c | n/c | n/c | -0.993 |
| OsIAA7 | Os02t0228900 | -1.164 | n/c | -0.849 | -0.58 | -0.756 | -1.054 |
| OsIAA8 | Os02t0723400 | -1.291 | n/c | n/c | -0.821 | -0.795 | -1.109 |
| OsIAA9 | Os02t0805100 | -1.807 | n/c | n/c | -0.876 | -0.249 | -1.817 |
| OsIAA10 | Os02t0817600 | -0.99 | n/c | n/c | n/c | n/c | -0.99 |
| OsIAA11 | Os03t0633500 | -1.503 | n/c | n/c | -0.769 | -0.109 | -1.633 |
| OsIAA12 | Os03t0633800 | -1.998 | -0.852 | -2.135 | n/c | n/c | -1.117 |
| OsIAA13 | Os03t0742900 | -1.572 | -0.17 | n/c | -0.217 | n/c | -1.741 |
| OsIAA14 | Os03t0797800 | -1.349 | n/c | n/c |  | -0.888 | -1.289 |
| OsIAA15 | Os05t0178600 | -1.247 | n/c | -0.816 | -0.86 | -0.837 | -1.051 |
| OsIAA16 | Os05t0186900 | -2.353 | n/c | -1.193 | -1.193 | -0.795 | -2.269 |
| OsIAA17 | Os05t0230700 | -1.509 | n/c | n/c |  | -0.338 | -1.674 |
| OsIAA18 | Os05t0523300 | -1.139 | n/c | -0.881 | -0.711 | 0.878 | -1.286 |
| OsIAA19 | Os05t0559400 | -1.384 | -0.873 | n/c | -0.104 | -0.872 | -1.35 |
| OsIAA20 | Os06t0166500 | -1.269 | n/c | -0.876 | -0.61 | -0.188 | -1.23 |
| OsIAA21 | Os06t0335500 | -0.845 | -0.874 | n/c | n/c | -0.635 | -0.548 |
| OsIAA22 | Os06t0355300 | -0.876 | - | 0.384 | n/c | -0.882 | -1.201 |
| OsIAA23 | Os06t0597000 | -0.883 | 1.907 | -0.848 | -0.668 | -0.873 | -0.959 |
| OsIAA24 | Os07t0182400 | 0.24 | n/c | n/c | n/c | n/c | 0.24 |
| OsIAA25 | Os08t0109400 | -1.383 | n/c | -0.88 | n/c | -0.851 | -1.19 |
| OsIAA26 | Os09t0527700 | -1.268 | -0.075 | -0.881 | n/c | n/c | -1.245 |
| OsIAA27 | Os11t0221000 | -1.058 | n/c | 0.632 | n/c | 0.058 | -1.303 |
| OsIAA28 | Os11t0221200 | -1.203 | - | -0.329 | -0.141 | -1.301 | -1.206 |
| OsIAA29 | Os11t0221300 | -1.664 | - | 0.095 | n/c | -1.021 | -1.828 |
| OsIAA30 | Os12t0601300 | -1.284 | n/c | n/c | n/c | 0.508 | -1.432 |
| OsIAA31 | Os12t0601400 | -1.728 | -0.327 | -1.392 | n/c | n/c | -1.483 |

n/c for seq without difference.

**Table S4.** Growth parameters of 7-day-old seedlings of wild-type (WT) plants and *Osiaa23* mutants.

| **Parameter/**  **Genotype** | **Kasalath**  **(WT)** | ***KaOsiaa23-3/-39*** | ***KaOsiaa23-3*** | ***KaOsiaa23-39*** | **Wuyunjing24**  **(WT)** | ***WuOsiaa23+1（g）/1（t）*** | ***WuOsiaa23+1（g）*** | ***WuOsiaa23+1（t）*** |
| --- | --- | --- | --- | --- | --- | --- | --- | --- |
| Number of (T1) plants | 10 | 10 | 4 | 6 | 15 | 15 | 8 | 7 |
| Shoot length (cm) | 15.44±0.76 | 9.28±0.49**^†^ | 13.70±0.65** | 7.22±0.41** | 18.37±0.75 | 16.50±0.75** | 14.04±0.67** | 14.77±0.52** |
| PR^†^ length (cm) | 4.29±0.42 | 2.63±0.31** | 3.95±0.44 | 2.48±0.28** | 4.33±0.28 | 3.67±0.27** | 3.51±0.21** | 3.56±0.37** |
| CR^†^ length (cm) | 14.62±0.48 | 6.60±0.47** | 9.93±0.43** | 4.48±0.31** | 15.17±0.44 | 6.95±0.54** | 6.49±0.70** | 6.80±1.23** |
| CR^†^ number | 7.3±0.7 | 5.8±1.0** | 7.0±0.8 | 4.7±0.5** | 6.6±0.8 | 3.4±0.5** | 3.0±0.8** | 3.4±0.5** |
| LR^†^ number | 49.4±4.4 | 0.0±0.0** | 46.0±5.4 | 0.0±0.0** | 46.0±4.1 | 42.5±2.1** | 42.1±2.6* | 42.0±4.1* |
| Fresh root weight (mg) | 10.81±0.32 | 5.79±0.44** | 8.33±0.53** | 4.61±0.44** | 16.01±0.64 | 9.06±0.70** | 8.56±0.83** | 7.44±0.66** |

^†^ PR: Primary Root; CR: Crown Root; LR: Lateral Root. Data represents the means±SD. * p<0.05, ** p<0.01 (Student’s t-test).

**Table S5.** Growth parameters of mature plants of wild-type (WT) plants and T1 mutants.

| **Parameter/**  **Genotype** | **Kasalath**  **(WT)** | ***KaOsiaa23-3/-39*** | ***KaOsiaa23-3*** | ***KaOsiaa23-39*** | **Wuyunjing24**  **(WT)** | ***WuOsiaa23+1（g）/1（t）*** | ***WuOsiaa23+1（g）*** | ***WuOsiaa23+1（t）*** |
| --- | --- | --- | --- | --- | --- | --- | --- | --- |
| Tiller number | 10.0±1.7 | 6.6±1.3**^†^ | 9.8±1.7 | 5.5±1.0** | 8.4±1.6 | 9.1±1.4 | 8.8±1.4 | 9.0±1.2 |
| Plant height (cm) | 129.7±2.4 | 101.9±3.2** | 125.5±2.9** | 92.5±2.3** | 96.5±2.1 | 85.1±1.8** | 84.2±1.0** | 84.6±1.5** |
| Ripening rate (%) | 95.2±1.8 | 19.3±3.3** | 90.8±1.6** | 6.4±2.9** | 78.7±4.1 | 74.8±2.2** | 73.2±3.6** | 72.9±5.7** |
| Growth cycle (day) | ~125 | ~125 | ~125 | ~125 | ~145 | ~145 | ~145 | ~145 |

^†^Data represents the means±SD. * p<0.05, ** p<0.01 (Student’s t-test).

**Table S6**. Splice junctions of OsIAA23 detected in root tissue of wild-type Wuyunjing24 and *WuOsiaa23* mutants.

| Sample | Junction | Junction_type | reads number | proportion 1^†^ | proportion 2^†^ |
| --- | --- | --- | --- | --- | --- |
| Wu-WT | chr06:23502267-23502353 | annotation | 602576 | 0.168812 | 0.110051 |
| *WuOsiaa23+1(g)/+1(t)* | chr06:23502267-23502353 | annotation | 339620 | 0.100128 | 0.080606 |
| *WuOsiaa23+1(g)* | chr06:23502267-23502353 | annotation | 441238 | 0.134850 | 0.079857 |
| Wu-WT | chr06:23502673-23502763 | annotation | 1869362 | 0.426398 | 0.453808 |
| *WuOsiaa23+1(g)/+1(t)* | chr06:23502673-23502763 | annotation | 1739011 | 0.459241 | 0.471878 |
| *WuOsiaa23+1(g)* | chr06:23502673-23502763 | annotation | 1831280 | 0.444940 | 0.468791 |
| Wu-WT | chr06:23502824-23502943 | annotation | 1779606 | 0.404506 | 0.433658 |
| *WuOsiaa23+1(g)/+1(t)* | chr06:23502824-23502943 | annotation | 1657337 | 0.440059 | 0.447075 |
| *WuOsiaa23+1(g)* | chr06:23502824-23502943 | annotation | 1739256 | 0.418832 | 0.449722 |
| Wu-WT | **chr06:23502686-23502763** | new | 584 | 0.000081 | 0.000202 |
| *WuOsiaa23+1(g)/+1(t)* | **chr06:23502686-23502763** | new | 1734 | 0.000558 | 0.000360 |
| *WuOsiaa23+1(g)* | **chr06:23502686-23502763** | new | 1125 | 0.000172 | 0.000410 |
| Wu-WT | **chr06:23502684-23502763** | new | 12 | 0.000000 | 0.000006 |
| *WuOsiaa23+1(g)/+1(t)* | **chr06:23502684-23502763** | new | 0 | 0.000000 | 0.000000 |
| *WuOsiaa23+1(g)* | **chr06:23502684-23502763** | new | 124 | 0.000011 | 0.000055 |
| Wu-WT | chr06:23502440-23502630 | new | 1517 | 0.000000 | 0.000768 |
| *WuOsiaa23+1(g)* | chr06:23502440-23502630 | new | 2394 | 0.000587 | 0.000607 |
| Wu-WT | chr06:23502427-23502576 | new | 2355 | 0.000002 | 0.001190 |
| *WuOsiaa23+1(g)* | chr06:23502427-23502576 | new | 0 | 0.000000 | 0.000000 |

^†^ Proportion 1 and 2 indicates the rate of the read number of corresponding junction reads to the total number of junction reads of PCR products respectively amplified by Primers OsIAA23-transcript –AlU1L and OsIAA23-transcript –A2U1L.

**Table S7**. Splice junctions of OsIAA23 detected in root tissue of wild-type Kasalath and *KaOsiaa23* mutants.

| Sample | Junction | Junction_type | reads number | proportion 1^†^ | proportion 2^†^ |
| --- | --- | --- | --- | --- | --- |
| Ka-WT | chr06:23502267-23502353 | annotation | 310048 | 0.124916 | 0.098578 |
| *KaOsiaa23-3/-39* | chr06:23502267-23502353 | annotation | 17094 | 0.026428 | 0.002040 |
| *KaOsiaa23-39* | chr06:23502267-23502353 | annotation | 19317 | 0.011225 | 0.000070 |
| Ka-WT | chr06:23502673-23502763 | annotation | 1299106 | 0.448731 | 0.461178 |
| *KaOsiaa23-3/-39* | chr06:23502673-23502763 | annotation | 599252 | 0.499464 | 0.506951 |
| *KaOsiaa23-39* | chr06:23502673-23502763 | annotation | 1332784 | 0.575014 | 0.506016 |
| Ka-WT | chr06:23502824-23502943 | annotation | 1229402 | 0.423787 | 0.436993 |
| *KaOsiaa23-3/-39* | chr06:23502824-23502943 | annotation | 573619 | 0.473811 | 0.489639 |
| *KaOsiaa23-39* | chr06:23502824-23502943 | annotation | 1262915 | 0.539234 | 0.493650 |
| Ka-WT | **chr06:23502686-23502763** | new | 740 | 0.000349 | 0.000203 |
| *KaOsiaa23-3/-39* | **chr06:23502686-23502763** | new | 511 | 0.000244 | 0.000617 |
| *KaOsiaa23-39* | **chr06:23502686-23502763** | new | 1208 | 0.000704 | 0.000000 |
| Ka-WT | **chr06:23502684-23502763** | new | 118 | 0.000000 | 0.000068 |
| *KaOsiaa23-3/-39* | **chr06:23502684-23502763** | new | 0 | 0.000000 | 0.000000 |
| *KaOsiaa23-39* | **chr06:23502684-23502763** | new | 0 | 0.000000 | 0.000000 |
| Ka-WT | chr06:23502078-23502612 | new | 4 | 0.000000 | 0.000002 |
| *KaOsiaa23-3/-39* | chr06:23502078-23502612 | new | 0 | 0.000000 | 0.000000 |
| *KaOsiaa23-39* | chr06:23502078-23502612 | new | 166 | 0.000000 | 0.000243 |
| Ka-WT | chr06:23502050-23502612 | new | 88 | 0.000000 | 0.000051 |
| *KaOsiaa23-3/-39* | chr06:23502050-23502612 | new | 0 | 0.000000 | 0.000000 |
| *KaOsiaa23-39* | chr06:23502050-23502612 | new | 0 | 0.000000 | 0.000000 |

^†^ Proportion 1 and 2 indicates the rate of the read number of corresponding junction reads to the total number of junction reads of PCR products respectively amplified by Primers OsIAA23-transcript –AlU1L and OsIAA23-transcript –A2U1L.
